# The Cost of a Locum: A Simulation to Determine When You Are Paying Too Much for Your Anesthesia Locum Tenens Coverage

**DOI:** 10.7759/cureus.58853

**Published:** 2024-04-23

**Authors:** James Cross, Yeshwanth Lolla, Chris Fichman, Michael Weingarten, Michael Howley

**Affiliations:** 1 Educational Affairs, Drexel University College of Medicine, Philadelphia, USA; 2 Marketing, Drexel University's LeBow College of Business, Philadelphia, USA; 3 Computer Engineering, NASA (National Aeronautics and Space Administration), Baltimore, USA; 4 Surgery, Drexel University College of Medicine, Philadelphia, USA

**Keywords:** statistical modeling, economic simulation, heath economics, locum tenens, anesthesia economics

## Abstract

Background: Current research on locum tenens physicians has primarily focused on their safety, reliability, and patient outcomes, leaving a significant gap in understanding the financial implications of their employment in health systems. Amidst a persistent shortage of physicians across specialties, healthcare organizations have increasingly relied on locum tenens to meet the rising demand for clinical services. This study aims to bridge the knowledge gap by evaluating the financial feasibility of employing locum tenens physicians compared to full-time anesthesiologists, given the context of growing physician shortages and increasing healthcare demands.

Methods: We developed a Python simulation model to compare the costs of hiring locum tenens versus full-time anesthesiologists. The model inputs included hourly rates for both locum tenens and full-time anesthesiologists and the upfront hiring costs for full-time physicians. By plotting these costs against each other, the model identifies the breakeven point: the number of working hours at which the cost of employing a locum tenens physician equals that of hiring a full-time physician. Utilizing Monte Carlo simulations with data from the Northeastern United States, we assessed the variability and determined an average breakeven point across different scenarios.

Results: The Monte Carlo simulation, based on 10,000 iterations, revealed an average breakeven point of 665 hours, corresponding to just over 11 weeks of 60-hour workweeks. This suggests that for any locum tenens engagement exceeding this duration, hiring a full-time anesthesiologist becomes more cost-effective for the healthcare institution. The simulation also showed that 28% of scenarios had a breakeven point below 60 days, highlighting the financial dynamics and decision-making complexities in employing locum tenens versus full-time physicians.

Conclusions: The findings indicate that employing locum tenens physicians for durations shorter than 665 hours remains financially viable compared to the option of hiring full-time anesthesiologists. However, the significant variability observed in the simulations underscores the importance of context in making staffing decisions. Healthcare organizations must consider the specific needs and circumstances of their operations when deciding between hiring locum tenens and full-time physicians, especially for longer-term coverage requirements.

## Introduction

One of the interesting phenomena that arose from the COVID-19 pandemic was the explosion of locum tenens offerings around the United States [1]. As hospitals and health systems found themselves underequipped to address the pandemic and short on doctors, they turned to offering temporary positions to physicians for large amounts of hourly pay [1]. Paralleling what was happening with travel nursing, physicians across all specialties were being offered temporary contracts that were well above what the market was offering prior [1,2]. This incentivized many physicians to leave their positions and pursue these lucrative offers. While COVID highlighted this style of employment, locum tenens have been around for decades. Despite their longevity, there is a striking paucity of research into the finances of hiring these temporary physicians.

Locum tenens is the concept historically applied by the Centers for Medicare and Medicaid Services to denote what is now known as a fee-for-time compensation arrangement [3]. In the case of a locum tenens, a substitute physician provides services in place of the regular physician, and the regular physician can bill Medicare for those services provided to Medicare [3,4]. Under this designation, the stand-in physician may use the primary physician’s Medicare identification number for a maximum of 60 days for billing [3].

Current research on locum tenens has looked at the safety, malpractice reliability, and outcomes of their usage [4,5]. Studies have shown that locum tenens doctors did not have a higher mortality rate, adverse event rate, or malpractice payout rate when compared to their non-locum peers [4-6]. However, no research has looked at the financial feasibility of their employment by health systems. Over the past several decades, there has been a provider shortage in physician specialties, which was greatly exacerbated by the pandemic [7]. The rates of locum tenens usage by healthcare organizations (HCOs) had been increasing leading up to the pandemic, and now over 85% of HCOs utilize locum tenens in some capacity [8-10]. While many of these positions are at remote sites that are historically difficult to staff regardless of incentives, larger HCOs near cities are beginning to rely on them as well [11,12]. These temporary physicians come with a price tag that can be double or triple the hourly pay of a full-time physician at the same institution [11,12]. As their use continues to increase, the question of how much these locum tenens are costing healthcare systems arises. At what point would it be more affordable for these larger HCOs to hire additional full-time physicians rather than rely on locum tenens?

The purpose of this article is to determine the point where an HCO is better off hiring a full-time physician rather than using locum tenens to fill in the gaps. Specifically, we will be looking at anesthesiologist locum versus full-time usage due to the availability of financial data. Despite their wide usage and popularity, there is almost no data regarding the finances of using locum tenens in practice due to the proprietary nature of the financial data needed to conduct such a project. Because of this, the wide-scale analysis that would be needed to determine the financial costs of locum tenens is not feasible. Instead, this research created a Monte Carlo simulation using national salary averages to find out the point at which the use of locum tenens physicians is more expensive compared to hiring a full-time physician.

## Materials and methods

We created a Monte Carlo simulation to determine the cost of hiring locum tenens versus hiring full-time anesthesiologists. For this project, anesthesiologist data was exclusively used due to the abundance of salary and locum tenens data available. A model was created in Python that takes inputs for the hourly rates of locum tenens anesthesiologists, full-time anesthesiologists, and the upfront cost required to hire a full-time anesthesiologist; it then plots them against each other. If all of this information is known, it can be entered into this model to find out the number of working hours at which the locum tenens’ higher hourly pay costs the hiring healthcare institution more money than onboarding a new full-time anesthesiologist. This point is called the breakeven point.

**Figure 1:**
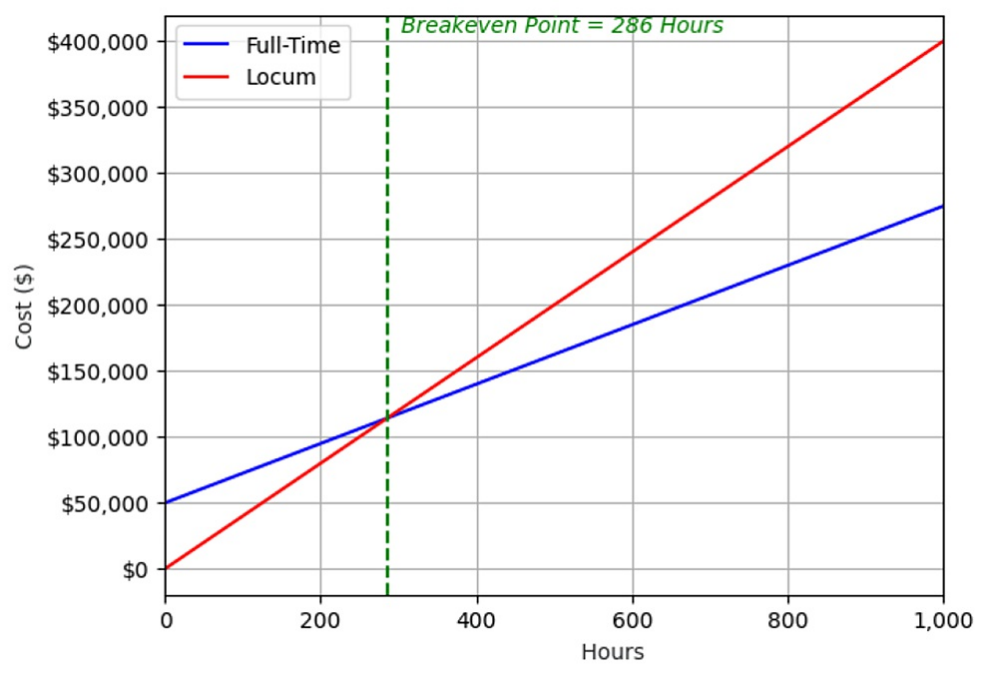
This example shows a single simulation of determining the breakeven point where all initial conditions are known (onboarding costs, full-time anesthesiologist cost, and locum tenens cost). The intersection of the two lines represents the point where hiring locum tenens becomes more costly than hiring an additional full-time anesthesiologist (the breakeven point). Assumptions include a full-time employee hourly rate of $225, a full-time employee onboarding fee of $50,000, and a locum employee hourly rate of $400.

For full-time anesthesiologists, the slope of this line is the combined value of the salary and benefits broken down into cost per hour, and the y-intercept is the cost to recruit and onboard a physician. For locum tenens, the slope is the hourly cost to hire the physician, and the y-intercept is zero since there is no initial cost. The intersection of these two plots represents the point where the costs of hiring a locum tenens versus a full-time physician are equal. At any point greater than that intersection, the cost of a full-time physician is less than that of the locum tenens (Figure 1).

This model would determine the breakeven point for comparing two individuals, but to determine what this breakeven point would be on a larger scale, we would need access to know how much hospitals were spending on hiring full-time physicians and how much they were paying for locum tenens. As this information was unavailable, we decided to use a Monte Carlo simulation to make an approximation of this data. For this simulation, all salary information was for providers in the Northeast region of the United States, which includes Pennsylvania, New York, New Jersey, Rhode Island, Connecticut, Massachusetts, New Hampshire, Vermont, and Maine. This was done to reduce the variability of salaries across regions of the United States because physician salary information is often reported based on regions in the United States.

Full-time anesthesiologists mean that salary and standard deviation were taken as a weighted average from three separate self-reported physician salary reports: The Bureau of Labor and Statistics, the Doximity, and the Association of American Medical Colleges (AAMC) Physician Specialty Data Report [13-15]. Initial hiring fees for full-time physicians were determined using estimated breakdowns posted by physician recruitment agencies [16]. Anesthesiologist locum tenens pay and standard deviations were taken from postings on GasWork.com, an anesthesiologist job listing site [17]. As locum tenens are paid hourly, the full-time anesthesiologist's salary and benefits were broken up into an hourly rate based on an average workweek of 60 hours to best compare the two groups [18]. The mean and standard deviation were determined for full-time anesthesiologist salary, onboarding costs for anesthesiologists, and locum tenens hourly pay in the Northeast.

These averages and standard deviations were used to create data points under a normal distribution. This process was repeated 10,000 times, simulating 10,000 data points for the cost of hiring a full-time anesthesiologist and the cost of hiring a locum tenens. With these data points, an average breakeven point was calculated along with the standard deviation. Additionally, we examined the percentage of the 10,000 simulations that had their breakeven point below 60 days, which is the maximum continuous time a locum tenens can be contracted.

## Results

**Figure 2:**
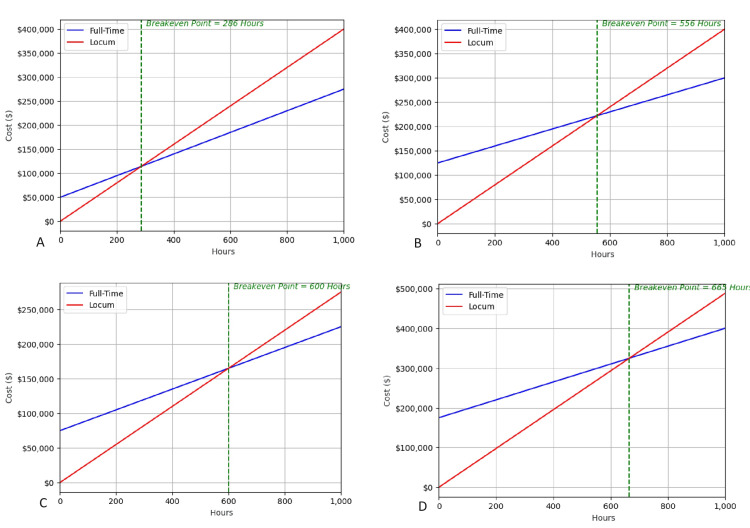
This is a sampling of four scenarios out of the 10,000. We start with a set of initial conditions based on average values to get a breakeven point. The Python program then changes the numbers to see how it affects the breakeven point. We re-run the scenarios 10,000 times, adjusting the values based on a normal distribution each time. This creates a final list of 10,000 breakeven points that are then analyzed. Here, we show a sampling of four simulations to illustrate how the parameters are changed each time. The breakeven point is denoted by the green line. Simulation A shows the breakeven point when the full-time salary is set at $200/hr, onboarding is set at $50,000, and locum tenens salary is set at $400/hr. Simulation B shows the breakeven point when the full-time salary is set at $150/hr, onboarding is set at $125,000, and locum tenens salary is set at $400/hr. Simulation C shows the breakeven point when the full-time salary is set at $174/hr, onboarding is set at $75,000, and locum tenens salary is set at $350/hr. Simulation D shows the breakeven point when the full-time salary is set at $165/hr, onboarding is set at $175,000, and locum tenens salary is set at $433/hr.

The Monte Carlo simulation was run 10,000 times (Figure 2) using the averaged values of full-time anesthesiologist pay, anesthesiologist onboarding costs, and locum tenens pay (Table 1).

**Table 1:** Financial information for full-time anesthesiologists, onboarding costs for new physicians, and anesthesiologist locum tenens rates in the Northeast United States Mean and standard deviations for full-time anesthesiologists were created by averaging out annual salaries from Physician Pay Reports [13], Doximity Salary Map [14], and AAMC Physician Specialty Data Report [15] for the Northeast region. The mean and standard deviation for onboarding costs for full-time anesthesiologists were taken from Weatherby Healthcare [16]. The mean and standard deviation for locum tenens were taken from a random sampling of job listings on GasWorks.com [17] for the Northeast region. AAMC: Association of American Medical Colleges.

	Annual full-time anesthesiologist salary + benefits	Hourly full-time anesthesiologist salary + benefits	Onboarding costs for full-time anesthesiologist	Locum tenens hourly cost
Mean	$467,200	$225.00	$90,000	$375
SD	$67,952	$32.60	$50,000	$25

In the analysis of the 10,000 simulations, we found the average breakeven point for employing locum tenens versus full-time anesthesiologists to be 665 hours, equivalent to just over 11 weeks for a 60-hour workweek (Table 2). This indicates that for any need exceeding 665 hours, it is more cost-effective for hospitals to hire a full-time anesthesiologist. Notably, 28% of these simulations had breakeven points under 60 workdays, which is the maximum time a locum tenens can work consecutively under another physician's license. This means in 72% of cases, opting for a full-time anesthesiologist becomes financially better after the locum tenens' working limit is reached.

**Table 2:** Results of the Monte Carlo simulation

Results of the Monte Carlo simulation	
Average breakeven time (hours)	665.2
Standard deviation (hours)	233.4
% of the time breakeven value below 60 days	28%

The results showed significant variability, with a standard deviation of 233.4 hours. This variability emphasizes the complexity of staffing decisions, which depend on factors like regional salary differences and specific demands of the specialty. The findings suggest that healthcare organizations need to consider their unique situations carefully when deciding between locum tenens and full-time hires.

## Discussion

The majority of locum tenens positions are for short-term coverage that falls well short of these ranges. However, there are situations where a healthcare organization could use this information for hiring decisions and financial best practices. First, 85% of healthcare organizations use locum tenens to meet the rising population demand [8]. Despite hiring locums for expected short-term coverage, many practices rotate through locum contracts to meet demand in their areas. These HCOs utilize loopholes in the locum tenens rules to allow them to work in the capacity of a full-time physician. This makes recruitment and retention much easier at the expense of higher pay.

The second is large HCOs or physician groups that employ many members of a specialty. Over one year, some of them will need to take a leave due to vacation, injury, maternity, paternity, and others. Locum tenens will be hired to cover these short-term absences [10]. Based on expected or historic absence data, an HCO could save money by hiring an additional full-time physician rather than relying on piecemeal locum tenens coverage. Ultimately, there is great variability in how and why locum tenens are hired, so it is important to take individual needs and context into any hiring decisions involving locum tenens.

Using a simulation for analysis presents inherent limitations that merit discussion. This process requires the assumption of certain conditions regarding the applied data to employ a simulated model effectively. Specifically, we assumed normal distributions for full-time anesthesiologist salaries, locum tenens compensation, and onboarding costs. The publicly available data on physician and locum tenens salaries exhibited considerable variance, which could impact the accuracy of our assumptions.

Our financial data was exclusively derived from sources in the Northeast region of the United States, without distinguishing between rural and urban settings. This lack of differentiation overlooks potentially significant discrepancies between these areas. Furthermore, the simulation specifically focuses on anesthesiologists in the Northeastern United States, limiting its applicability to other specialties or regions. A comparative analysis of breakeven points across different specialties and regions might reveal noteworthy trends. Notably, locum tenens positions are predominantly offered at community hospitals, as opposed to academic centers. This setting tends to prioritize simpler and quicker procedures over the more complex and lengthy cases typically seen in larger hospitals.

A collaboration with a large healthcare organization, granting access to comprehensive financial data on full-time and locum tenens staffing, could significantly enhance the accuracy and utility of our simulated model. The primary limitation of this project stems from its reliance on generalized national salary averages rather than detailed, real-world data. Obtaining more precise data would greatly improve the simulation's reliability and relevance.

## Conclusions

Our research reveals that employing locum tenens physicians for over 665 hours becomes less cost-effective than hiring full-time anesthesiologists, based on a Monte Carlo simulation focusing on the Northeast United States. This breakeven point, equivalent to just over 11 weeks for 60-hour workweeks, underscores the financial viability of locum tenens for short-term needs. However, given the significant variability observed, healthcare organizations must tailor their staffing decisions to their specific situations. This study emphasizes the importance of context in choosing between locum tenens and full-time hires, suggesting that for longer-term needs, investing in full-time staff might be more economical.
